# The Airway Microbiome and Metabolome in Preterm Infants: Potential Biomarkers of Bronchopulmonary Dysplasia

**DOI:** 10.3389/fped.2022.862157

**Published:** 2022-05-10

**Authors:** Qi Xu, Jialin Yu, Dong Liu, Qi Tan, Yu He

**Affiliations:** ^1^Department of Pediatrics, Southern University of Science and Technology Hospital, Shenzhen, China; ^2^Children’s Hospital, Capital Institute of Pediatrics, Beijing, China; ^3^Department of Neonatology, Shenzhen People’s Hospital, Shenzhen, China; ^4^Department of Neonatology, Children’s Hospital of Chongqing Medical University, Chongqing, China

**Keywords:** BPD, microbiome, genomics, metabonomic, biomarkers

## Abstract

**Objectives:**

We investigated the genomic and metabolic characteristics of the airway microbiome in mild, moderate, severe, and non-bronchopulmonary dysplasia (BPD) preterm infants and explored possible mechanisms underlying BPD.

**Methods:**

Twenty-eight preterm infants with gestational age ≤34 weeks and intubated within 24 h after birth were enrolled. According to the severity of BPD, the patients were divided into mild, moderate and severe BPD groups, and the non-BPD group was the control group. Tracheal aspirates (TA) were obtained at intubation and on day 7 after birth. The bacterium in the aspirates were sequenced by 16S rRNA, and the metabolomics of the aspirates were identified by high performance liquid chromatography-quadrupole time of flight mass spectrometry (UHPLC-Q-TOF/MS). The correlation between the differential metabolite and differential bacteria was investigated using Pearson’s correlation coefficient corrected for gestational age and birth weight and Kyoto Encyclopedia of Genes and Genomes (KEGG) databases.

**Results:**

There were significant differences in the diversity and composition of airway microbiome and metabolome between severe, moderate and mild BPD and non-BPD premature infants. At birth (day 1), the difference was more pronounced than at day 7. The diversity of airway microbial community decreased, the abundance of *Stenotrophomonas* increased, and the increased level of sn-glycerol 3-phosphoethanolamine was positively correlated with the severity of BPD. There was a significant positive correlation between the abundance of *Stenotrophomonas* and the level of sn-glycerol 3-phosphoethanolamine.

**Conclusion:**

Decreased diversity of the airway microbiome, increased abundance of *Stenotrophomonas*, and increased level of sn-glycerol 3-phosphoethanolamine may have potential as biomarkers for BPD. The occurrence and severity of BPD are closely related to *Stenotrophomonas*, which may influence the composition of the lower airway microbiome through its metabolite sn-glycerol 3-phosphoethanolamine, and may be the triggering factor of the disease. The causal relationship needs further study.

## Introduction

Bronchopulmonary dysplasia (BPD) is a serious disease associated with premature that affects an estimated 50% of infants born at <28 weeks of gestation (1). Infants with BPD have an increased risk of mortality during the first year. Those who survive may suffer from long-term pulmonary impairment and abnormal neurodevelopment, which can result in substantial healthcare resource utilization and cost (2). Risk factors for BPD include gestational age at birth, impaired growth for gestational age, low infant birth weight, infectious exposures, barotrauma, oxygen exposure, and environmental cigarette smoke and the like (3). Evidence from epidemiological data, clinical data, and animal models indicates a key role for the microbiome in lung disease (4–6), and indicates that the airway microbiome is altered in multiple respiratory disorders (7, 8). Remote et al. found that intranasal lung inoculation with CNCM I 4970 aggravated asthma symptoms, while inoculation with TH1-promoting CNCM I 4969 had a protective effect, demonstrating that appropriate pulmonary bacterial stimulation early in life is crucial for young people prone to allergic asthma and may affect the development of allergic asthma (4). Some reports show that the airway microbiome is present at birth, and microbial dysbiosis may be associated with BPD (9–12). However, so far, there are few relevant studies and the results varied (9–12). Wagner et al. proposed that longitudinal changes in the airway microbial communities of preterm infants on mechanical ventilators may be related to the severity of BPD, whereas cross-sectional analysis of airway ecology at 7 days of age did not reveal an association with BPD severity (10). While Lal and his colleagues found the airway microbiome difference between BPD infants and control was observed at birth (9). Lohmann et al. concluded that reduced diversity of the microbiome may be an important factor in the development of BPD (11). In Wagner’s study (10) and Mourani’s study (12), *Staphylococcus* and *Ureaplasma* were the most the most common dominant organisms in the respiratory tract of infants. However, Lohmann et al. found that *Acinetobacter* was the predominant genus in the respiratory tract of infants at birth (11). In Wagner’s research, the cross-sectional dataset included only samples collected between 5 and 9 days of age among mild, moderate and severe BPD groups, microbes at birth are not tested and compared, and the non-BPD group was absent in this study (10). Lal’s investigation (9) and Lohmann’s study (11) complemented Wagner’s research, but they only compared the difference between the BPD and non-BPD groups, and further analysis of the relationship between disease severity and airway microbiome is needed. Preterm infants also have different dominant organisms in their airways (9–12). Based on their studies, we divided the enrolled infants into four groups (severe BPD, moderate BPD, mild BPD, and non-BPD) according to the severity of BPD and compared them at birth and at 7 days after birth. We also explored possible mechanism. Gut microbiota has been implicated in a variety of human diseases through its metabolites (13). Acevedo et al. proposed that microbiota is able to leave immune signals in the host via its metabolites (acetate, propionate, butyrate, and polyphenols), including those associated with allergies (14). Bacteria could produce metabolites that interact with the host and alter the development and progression of chronic respiratory diseases (15). Therefore, we hypothesized that airway microbial metabolism might play a role in the pathogenesis of BPD.

In this prospective observational cohort study, tracheal aspirates (TA) were collected during mechanical ventilation of infants to investigate: (1) airway microbiota at birth (Day 1) and on Day 7 after birth; (2) airway metabolomics characteristics at birth and on Day 7 after birth; and (3) the relationship between differential metabolites and specific bacteria in infants with severe BPD, moderate BPD, mild BPD, and non-BPD.

## Methods

This prospective observational cohort study was conducted at the Neonatal Intensive Care Unit of the Children’s Hospital of Chongqing Medical University between October 2017 and July 2018. The Institutional Review Board of Chongqing Medical University approved the protocol. Informed consent was received from the parents or guardians of all participants. The study was performed in accordance with approved guidelines.

### Patient Population and Clinical Data Collection

Infants born at <34 weeks gestation that underwent endotracheal intubation and mechanical ventilation in the first 24 h of life were included in this study. Exclusion criteria were: (1) clinical evidence of congenital heart disease [except patent ductus arteriosus (PDA), patent foramen ovale (PFO), or atrial septal defect (ASD) <1 cm, or ventricular septal defect (VSD) <2 mm if known prior to enrollment]; (2) lethal congenital abnormality; (3) congenital sepsis; (4) evidence of pulmonary hypoplasia; or (5) futile cases (anticipated death prior to hospital discharge) (10).

Infants were divided into four groups stratified by the diagnosis and severity of BPD: severe BPD, moderate BPD, mild BPD, and non-BPD. BPD was diagnosed based on the need for supplemental oxygen at 28 days of age (16, 17). BPD status and severity was assessed at 36 weeks postmenstrual age or 56 days after birth or upon discharge from hospital according to the National Institutes of Health workshop definition (17). Late onset sepsis was defined as a positive blood culture after 72 h of life.

Clinical data were collected from a review of electronic medical records at study enrollment and during hospitalization. Information on maternal history, delivery, and clinical assessments was recorded.

### Sample Collection

Tracheal aspirates (TA) were collected during mechanical ventilation at birth (Day 1) and on Day 7 after birth according to a previously published protocol (11, 12, 17). Briefly, 0.5 ml of sterile isotonic saline was instilled into the infants’ endotracheal tubes. Infants were manually ventilated through their endotracheal tube for three breaths using a bag-mask, and fluid was suctioned into a sterile mucus trap (11). Samples were divided into 2 aliquots for extraction of bacterial DNA or metabolomics research and frozen at –80^°^C until further processing.

### Isolation of Microbial DNA, Creation of 16S V4 Amplicon Library, and DNA Sequencing

Microbial genomic DNA from each sample was isolated and purified. The V4 region of the 16S rRNA gene from the microbial DNA was amplified using a polymerase chain reaction (PCR) with unique barcoded primers to create an “amplicon library” (18). The library was sequenced using the Illumina MiSeq platform and subsequently quantified (KAPA Library Quantification Kit KK4824), according to the manufacturer’s instructions. Sequence data presented in the study are publicly available. This data can be found here: http://www.ncbi.nlm.nih.gov/sra/PRJNA800242.

### Profiling of 16S rRNA Gene Sequencing Data

Using the Quantitative Insights into Microbial Ecology (QIIME) 1.8.0 pipeline, the raw sequences were processed to concatenate reads into tags according to the overlapping relationship. Reads from each sample were separated with barcodes, and low-quality reads were removed. Processed tags were clustered at 97% similarity into operational taxonomic units (OTUs). The OTUs were assigned to taxa by matching to the Greengenes database (Release 13.8). Alpha diversity analyses (Shannon index) and beta diversity analyses [principal coordinate analysis (PCoA)] were performed. Comparison of microbiome composition among the four groups was performed using one-way ANOVA, and the differences of microbiome were considered statistically significant with a *P*-value of < 0.05.

### Metabolomics Analysis Based on UHPLC-Q-TOF/MS

TA samples were analyzed using an ultra-high-performance liquid chromatography (UHPLC) system (1290 Infinity LC, Agilent Technologies, Palo Alto, CA, United States) coupled to a quadrupole time-of-flight mass spectrometer (AB Sciex TripleTOF 6600, Framingham, MA, United States) at Shanghai Applied Protein Technology Co., Ltd.

Samples were thawed at 4°C and 100 μL aliquots were mixed with 400 μL of cold methanol/acetonitrile (1:1, v/v) to remove the protein. After centrifuging for 15 min (14,000 g, 4°C), the supernatant was dried in a vacuum centrifuge. For liquid chromatography-mass spectrometry (LC-MS), samples were dissolved in 100 μL acetonitrile/water (1:1, v/v). Pooled quality control (QC) samples were used to monitor the stability and repeatability of instrument analysis. The QC samples were inserted regularly and analyzed in every five samples.

Raw LS electrospray ionization (ESI) MS data were converted into m/z format and analyzed for non-linear retention time (RT) alignment, peak detection, and filtration.

### Profiling of Metabolomics Data

Processed data were normalized to total peak intensity, imported into SIMCA-P (version 14.1, Umetrics, Umea, Sweden), and analyzed using Pareto-scaled principal component analysis (PCA). One-way ANOVA was used to determine the significance of each metabolite with a VIP value > 1. *P* < 0.05 were considered statistically significant. Discriminatory metabolites within the data set were visualized as heat maps, which were generated using a hierarchical clustering algorithm. Molecules associated with significant changes were searched against the Kyoto Encyclopedia of Genes and Genomes (KEGG) database.^1^

### Statistical Analysis

Statistical analysis was performed using SPSS version 22.0 for Windows (SPSS Inc., United States). Normally distributed data are expressed as mean ± SD; non-normally distributed data are expressed as median and interquartile range (IQR). Between group differences were analyzed with Fisher’s Exact test for categorical variables and Kruskal-Wallis test for continuous variables after subsampling. Measurement data will also be compared by means of univariate analysis. We deal with the abnormal value by Pauta criterion (19). Correlations between microbiome–related metabolites and bacterial species were evaluated using Pearson’s correlation coefficient corrected by gestational age and birth weight. *P* < 0.05 was considered statistically significant.

## Results

### Demographic and Clinical Characteristics of the Enrolled Patients

In this study, 28 premature infants were divided into 4 groups, including 8 severe BPD, 5 moderate BPD, 10 mild BPD, and 5 non-BPD. The demographic and clinical characteristics of the included infants are shown in Table 1. There were no significant differences in the demographic and clinical characteristics between the groups, except for number of hours on mechanical ventilation and days on antibiotics.

**TABLE 1:** Demographic and clinical characteristics of the included patients.

	Severe BPD group (*n* = 8*)	Moderate BPD group (*n* = 5)	Mild BPD group (*n* = 10*)	Non-BPD group (*n* = 5*)	*P-*value
Gestational age (mean ± SD)	30.28 ± 2.22	32.54 ± 1.35	31.76 ± 1.54	31.57 ± 0.61	0.092
**GA categories**					
≤28 weeks, n (%)	3 (37.5%)	0 (0%)	1 (10%)	0 (0%)	0.090
28–32 weeks, n (%)	3 (37.5%)	1 (20%)	4 (40%)	4 (80%)	0.280
>32 weeks, n (%)	2 (25%)	4 (80%)	5 (50%)	1 (20%)	0.428
Birth weight (g) (mean ± SD)	1335.75 ± 380.95	1530.0 ± 363.66	1574 ± 211.72	1640 ± 139.10	0.266
1 min Apgar (mean ± SD)	4.25 ± 2.96	5.00 ± 4.06	5.4 ± 2.72	7.00 ± 2.24	0.372
5 min Apgar (median)	8	8	8.5	9	0.722
10 min Apgar (median)	8.5	8	9	10	0.120
Male gender, n (%)	4 (50%)	1 (20%)	6 (60%)	4 (80%)	0.249
Han population, n (%)	8 (100%)	4 (80%)	10 (100%)	5 (100%)	0.305
Cesarean delivery, n (%)	5 (62.5%)	5 (100%)	8 (80%)	5 (100%)	0.128
Antenatal steroids, n (%)	6 (75%)	2 (40%)	7 (70%)	3 (60%)	0.610
Rupture of membranes > 18 h, n (%)	1 (12.5%)	1 (20%)	1 (10%)	1 (20%)	0.934
Women given antibiotics before delivery, n (%)	1 (12.5%)	0 (0%)	1 (10%)	1 (20%)	0.973
Intrauterine growth restriction, n (%)	1 (12.5%)	1 (20%)	0 (0%)	0 (0%)	0.337
Treatment with surfactant, n (%)	6 (75%)	2 (40%)	8 (80%)	3 (60%)	0.440
Mechanical Ventilation hours (mean ± SD)	620 ± 215	231 ± 148	353 ± 260	148 ± 45	0.002
Antibiotic days (mean ± SD)	43 ± 21	22 ± 16	23 ± 12	13 ± 5	0.009
Pneumonia, n (%)	10 (100%)	5 (100%)	9 (90%)	5 (100%)	0.547
Pulmonary hemorrhage, n (%)	4 (50%)	2 (40%)	3 (30%)	0 (0%)	0.163
Late onset sepsis, n (%)	4 (50%)	0 (0%)	5 (50%)	1 (20%)	0.088
Necrotizing enterocolitis ≥ stage 2, n (%)	0 (0%)	0 (0%)	1 (10%)	0 (0%)	0.547

*One infant lost Day 1 specimen but had Day 7 specimen, so the number of 16S rRNA gene sequencing and metabolomic analyses is less than the number of patients.

### Diversity and Composition of the Airway Microbiome

**FIGURE 1:**
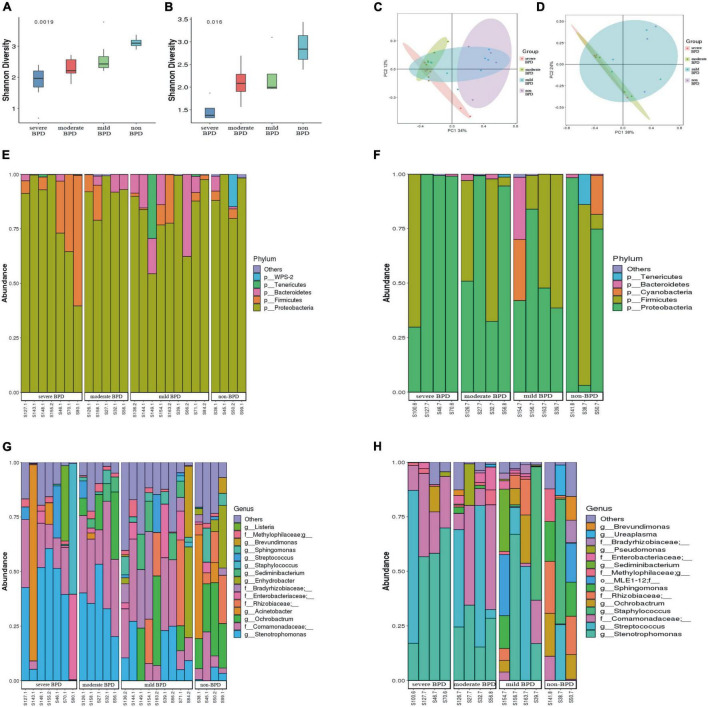
Diversity and composition of the airway microbiome. **(A)** Shannon index at birth (Day 1); **(B)** Shannon index on Day 7 after birth (a greater Shannon index is indicative of higher microbial diversity); **(C)** Principal coordinate analysis (PCoA) of microbial communities at birth (Day 1); **(D)** PCoA of microbial communities on Day 7 after birth (samples near to each other have similar microbial composition, while samples far from each other have distinct microbial composition); **(E,F)** Relative abundance of bacterial phyla (**E** stands for day 1 after birth, **F** stands for day 7 after birth). **(G,H)** Relative abundance of bacterial genera (**G** stands for day 1 after birth, **H** stands for day 7 after birth). (On day 1, the number of infants with severe BPD, moderate BPD, mild BPD and non-BPD was 7, 5, 9, and 4, respectively. On day 7, the number of babies was 4, 4, 4, and 3, respectively. On day 7, some specimens were missing).

The Shannon index was significantly lower at birth (Day 1) (*P* = 0.0019; Figure 1A) and on Day 7 after birth (*P* = 0.016; Figure 1B) in infants with BPD compared to non-BPD. The difference was more pronounced on Day 1 and was negatively correlated with the severity of BPD. The Shannon index was further analyzed by ANOVA way (Dunnett test), non-BPD group was control group. For Day 1, difference between severe BPD group and non-BPD group was significant (*P* = 0.001), the difference between moderate BPD group and non-BPD group was also found (*P* = 0.039), however, there was no difference between mild BPD group and control group (*P* = 0.196). For Day 7 after birth, the difference between severe BPD group and non-BPD group was found (*P* = 0.006), however the *P*-value for moderate BPD group vs. non-BPD group and mild BPD group vs. control group was 0.111 and 0.226, respectively. Principal coordinates analysis (PCoA) also showed a significant difference in the bacterial composition of the airway microbiome at birth (Day 1) between the four groups (Figure 1C), and a less distinct difference on Day 7 after birth (Figure 1D).

**FIGURE 2:**
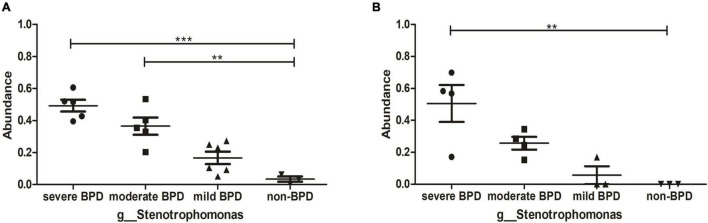
Abundance of *Stenotrophomonas*. **(A)** The abundance of *Stenotrophomonas* at birth (Day 1); **(B)** The abundance of *Stenotrophomonas* on Day 7 after birth. ***P < 0.001; **P < 0.01. On day 1, the number of infants with severe BPD, moderate BPD, mild BPD, and non-BPD was 5, 5, 6, and 3, respectively. On day 7, the number of infants was 4, 4, 3, and 3, respectively.

At the phylum level, *Proteobacteria* was dominant in the airway microbiome of all infants at birth (Day 1) (Figure 1E) and on Day 7 after birth (Figure 1F), and there were no significant differences in the composition of the airway microbiome among the four groups. At the genus level, the composition of the airway microbiome was significantly different among the four groups at birth (Day 1). *Stenotrophomonas* was more abundant in BPD compared to non-BPD, and abundance was positively correlated with the severity of the disease (*P* < 0.05) (Figure 2A). But the results on Day 7 after birth were similar and not statistically significant (*P* = 0.064) (Figure 2B). Then taking non-BPD group as control group, the three disease groups were compared to control group by ANOVA way analysis (Dunnett test), respectively. For 1 day, the *P*-value for severe BPD group vs. non-BPD group, moderate BPD group vs. non-BPD group and mild BPD group vs. control group was 0.000, 0.001, 0.140, respectively. And on day 7 the *P*-value was 0.002, 0.090, and 0.921, respectively.

### Metabolomics Analysis of Tracheal Aspirates

**FIGURE 3:**
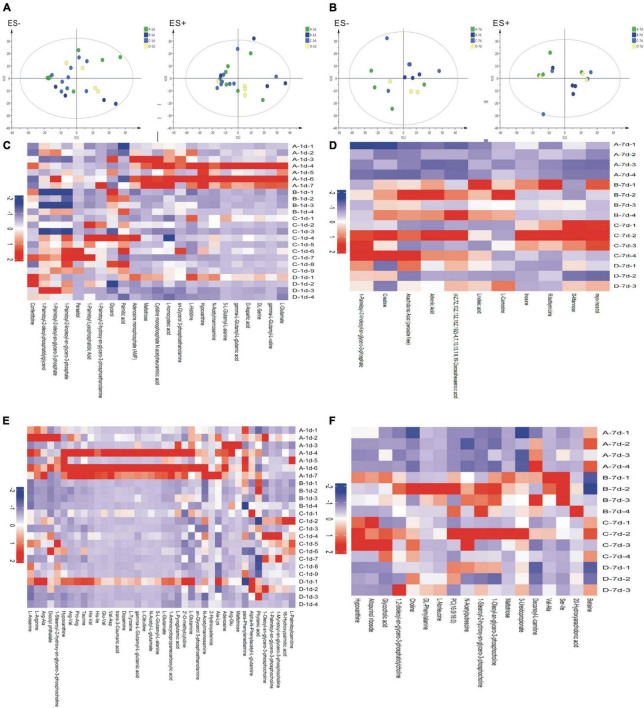
Metabolic profiles. **(A,B)** Principal component analysis (PCA) based on the metabolic profiles in sputum samples (**A** stands for day 1 after birth, **B** stands for day 7 after birth); **(C–F)** Hierarchical clustering heat maps showing patterns in molecular data across groups. The relative amounts of the 86 compounds were transformed into Z scores (**C** stands for day 1 after birth and ES-, **D** stands for day 7 after birth and ES−, **E** stands for day 1 after birth and ES+, **F** stands for day 7 after birth and ES+) (ES+: positive ion mode, ES-: negative ion mode) (A, severe BPD; B, moderate BPD; C, mild BPD; D, non-BPD). On day 1, the number of infants in A, B, C, D group was 7, 4, 9, and 4, respectively. On day 7, the number of infants in A, B, C, D group was 4, 4, 4, and 3, respectively.

**FIGURE 4:**
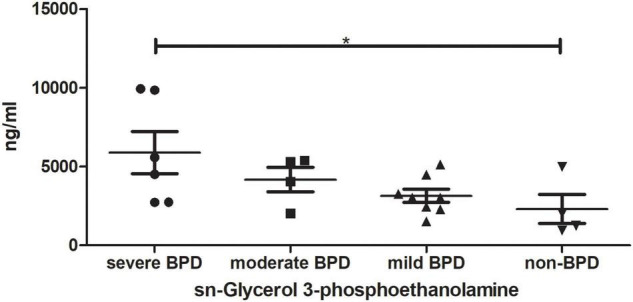
sn-Glycerol 3-phosphoethanolamine level at birth (Day 1). *P < 0.05 (sever BPD group: *n* = 6; moderate BPD group: *n* = 4; mild BPD group: *n* = 8; non-BPD group: *n* = 4).

TAs were subjected to LC/MS analysis in positive ion mode (ES+) and negative ion mode (ES-). Principle component analysis (PCA) was performed to reduce dimensionality in the dataset (Figures 3A,B). Parameters of the multivariate models were supplied in Supplementary Table 1. One-way ANOVA analysis result was supplied in Supplementary Table 2. Hierarchical clustering heat maps visualized patterns in molecular data across groups (Figures 3C–F). There were significant differences in 63 metabolites, including 23 in ES- and 40 in ES+ (Figures 3C,D), between the four groups at birth (Day 1), and 29 metabolites, including 11 in ES- and 18 in ES+, on Day 7 after birth (Figures 3E,F). Among these metabolites, sn-glycerol 3-phosphoethanolamine was positively correlated with BPD severity at birth (Day 1) (Figure 4), but not on Day 7 after birth. The level of sn-glycerol 3-phosphoethanolamine was high in sever BPD group, gradually decreased as the disease gets milder and was lowest in non-BPD group. The level of sn-glycerol 3-phosphoethanolamine in sever BPD group, moderate BPD group and mild BPD group was compared to non-BPD group respectively, and the difference was only found between sever BPD group and non-BPD group.

### Correlation Between the Airway Microbiome and Metabolites

**FIGURE 5:**
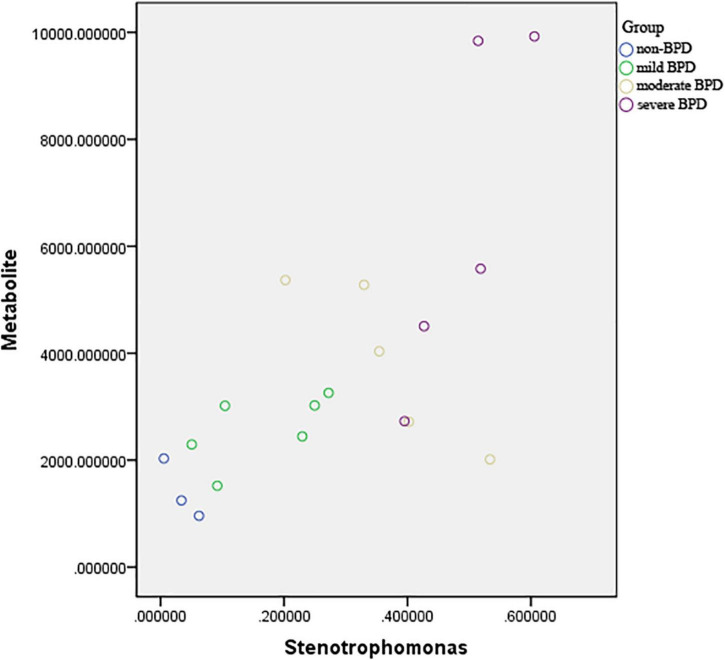
Scatter plot of the association between abundance of *Stenotrophomonas* and sn-glycerol 3-phosphoethanolamine level. *r* = 0.629, *P* = 0.007 (sever BPD group: *n* = 5; moderate BPD group: *n* = 5; mild BPD group: *n* = 6; non-BPD group: *n* = 3).

Pearson’s correlation coefficient corrected by gestational age and birth weight was used to explore the functional correlation between the changes in the airway microbiome and differences in metabolites across the four groups at birth (Day 1) (Figure 5). There was a significant positive correlation between the abundance of *Stenotrophomonas* and sn-glycerol 3-phosphoethanolamine levels (*r* = 0.629, *P* = 0.007).

## Discussion

Studies on the correlation between infant BPD and the airway microbiome are few and the results are not consistent (9–12), and the clinical needs for characteristics of the airway microbiome and metabolomics in patients with BPD remain unmet. These results may contribute to the early detection of microbial biomarkers for BPD and further understanding of the pathophysiology of BPD. In our study, gestational ages and birth weights were higher than in other studies (5, 20, 21) due to different subjects. First, we excluded infants who died early in our study, but others did include infants who died early in their studies (19). Secondly, different regions and ethnicity may also have some influence.

Preterm birth is a strong predictor of BPD, with an inversely proportional incidence and severity (22, 23). In order to better analyze the effect of gestational age on this study, we stratified the gestational age groups and divided them into three groups according to gestational age: ≤28 weeks, 28–32 weeks, and >32 weeks. The classification of gestation age refers to Seth’s study (24) and Viscardi’s study (25). There was no statistical difference in gestational age between the four groups including the BPD groups and non-BPD group. For each gestation age group, there was no statistic difference too. In our study, we also found a negative correlation between gestational age and disease severity, as most of extremely preterm infants were in the severe BPD group. Although the scale of our study was small, there was no statistical difference in gestational age among the four groups, which reduced the statistical errors and biases. Intrauterine growth restriction (IUGR) is known to increase the risk of BPD (23). It is postulated that the biological mechanisms leading to IUGR, such as placental dysfunction, insulin growth factor, vascular endothelial growth factor (VEGF) and VEGF receptor deficiency, might also lead to fetal lung growth restriction (26). In our study, due to the small sample size, the number of infants with IUGR in each group was very small. There was 1 infant with IUGR in both sever and moderate BPD group, no infant with IUGR in mild and non-BPD groups. However, the proportion of IUGR in this study was consistent with others study (27). In Sheth’s research, the rate of IUGR was 10.5% (27), while in our study, the rate was 7.1%, the difference was not statistically significant. What’s more, there was no significant difference in IUGR between the four groups. Therefore, IUGR may not play a confounding role in this study. For maternal antibiotics use before delivery, no difference was found among four groups. Most mothers almost not accepted antibiotics treatment before delivery which was consistent with Qu’s research (28). Second, despite the negative effects of infection on lung development, there is no clear evidence that antibiotic therapy improves the respiratory course in these infants (29). So the influence of maternal antibiotics use before delivery may be very little to this research.

Prenatal steroid use was 40–75%, probably lower than in other studies (19, 27), where steroid use was 50–76%. Steroid use is low for a reason. First, some premature babies are unpredictable, and these infants miss out on prenatal steroids, which is the main reason. Secondly, some patients come from remote areas and do not often receive regular pregnancy tests and treatment.

Our results showed that multiple bacterial taxa can be identified in the respiratory secretions from intubated premature infants, even at birth and prior to surfactant administration. The diversity and composition of the airway microbiome at birth differs between infants with and without BPD, and the differences became less pronounced by Day 7 of life. Consistent with a previous report (11), our study showed that infants who would develop BPD had a lower diversity of airway microbiome. Our results also revealed that alpha diversity of airway biome may be negatively correlated with the severity of BPD. This phenomenon was more pronounced early in life. It follows that the microbiome might affect infants at an early age, and we should take action as early as possible. These results suggest that bacterial communities might play a role as promoters in the occurrence and development of BPD.

At the phylum level, there were no significant difference in the composition of the airway microbiome in infants with, without BPD and with BDP severity. *Proteobacteria* were the most abundant microbes in all infants. These data align with a previous report (11), but differ from another report that found *Staphylococcus* and *Ureaplasma* were the dominant microorganisms in infants’ airways at their facility (10). These disparate findings might be due to different environments, which likely influenced the composition of the airway microbiome. As suggested by Yang K and Dong W (30), that conflicting results related to the populations change at the phylum level might indicate that lung microbiota is affected by multiple factors, including demographic characteristics, geographic position, living environment, methods and detection reagents, and sequencing platforms, etc. In this study, the Illumina MiSeq platform was used to detect the respiratory microflora of premature infants in western China. At the genus level, the number of *Stenotrophomonas* in infants with BPD was significantly higher than that in ones without BPD, and the number of *Stenotrophomonas* was positively correlated with the severity of BPD. These results suggest that the diversity of bacteria in the airway microbial community of intubated premature infants decreased, while the abundance of *Stenotrophomonas* increased, which could be used as a microbial marker for the early detection of BPD in western China.

*Stenotrophomonas* is a nosocomial opportunistic pathogen in the *Xanthomonadaceae* family (31). *Stenotrophomonas* isolated from the environment or clinical setting exhibits resistance to antibiotics and stress and forms biofilms on various surfaces, including the abiotic surfaces of catheters and prosthetic devices (32, 33). The colonization of *Sterotrophomonas* in the lungs of patients with cystic fibrosis and immunocompromised patients may be a marker of chronic lung disease (32, 34–36). *Stenotrophomonas* can influence the spatial organization and thus affect the function and composition of complex microbiome (37, 38). Data from the present study suggest that *Stenotrophomonas* is closely related to the occurrence of BPD in intubated premature infants, and the causal relationship remains to be further explored.

Since the composition of the airway microbiome in preterm infants varies with the presence or absence of BPD and the severity of BPD, we characterized the airway metabolome of these infants. The results showed that 63 metabolites at birth (day 1) were significantly different and 29 metabolites at day 7, indicating that the changes of metabolites were in parallel with the changes of airway microbiome. Among these metabolites, sn-glycerol 3-phosphoethanolamine content was positively correlated with the severity of BPD, which might be a potential metabolic biomarker for the early detection of BPD. The KEGG database showed that sn-glycerol 3-phosphoethanolamine has a role in glycerophospholipid metabolism. Glycerophospholipid has structural functions in bacteria, facilitates bacterial adaptation to environmental conditions, and is involved in bacteria–host interactions (39). Glycerophospholipid is also associated with the pathophysiology of chronic obstructive pulmonary disease (COPD) (40). These findings suggest that sn-glycerol 3-phosphoethanolamine might affect airway microbiome composition and respiratory health in preterm infants. Our findings also identified a significant positive correlation between the abundance of *Stenotrophomonas* and the level of sn-glycerol 3-phosphoethanolamine, which has been confirmed by the KEGG database. Although the causal link between lung microbiota and BPD has not been fully demonstrated, it could be hypothesized that the lung maladaptive microbiome is a potentially harmful factor and driver in this disease (41). In this study, we found that the airway microbiota of infants with BPD is rich in *Stenotrophomonas*, which might be related to the production of sn-glycerol 3-phosphoethanolamine, which may be a pathogenic signal of these patients.

Most study just compared the BPD group with non-BPD group (26, 42, 43), in our study, the infants were more carefully divided into four groups, severe BPD, moderate BPD, mild BPD, and non-BPD. And we were lucky to find something that were related to the severity of the disease. First, there were significant differences in the diversity and composition of the airway microbiome and metabolome in preterm infants with severe, moderate, or mild BPD or non-BPD; and the differences were more pronounced at birth (Day 1) than on Day 7 of life. Secondly, decreased diversity of the airway microbiome, increased abundance of *Stenotrophomonas*, and increased level of sn-glycerol 3-phosphoethanolamine were positively associated with the severity of BPD, and might have potential as biomarkers for BPD. Thirdly, the abundance of *Stenotrophomonas* had a significant positive correlation with sn-glycerol 3-phosphoethanolamine level.

However, there were several limitations to this study. First, the sample size was small owing to very few preterm infants could obtain both 1- and 7-day specimens. But this study is still going on. More data will be reported at a later stage. Second, conclusions might not be extended to other populations, and only applies to infants with high incidence of BPD, such as low gestational age at birth, low birth weight, infectious diseases, etc. Third, the data provide no evidence that the airway microbiome directly causes BPD. This is just a prospective observational cohort study, in the near future validation experiments will be carried out. In addition to these, nearly all of the infants in the study were treated with antibiotics for more than 7 days. Only one infant was treated with antibiotics for 5 days. Therefore, the effect of antibiotics on the enrolled infants may be the same at the first week of life. While there was difference over the total number of days of antibiotic use, the effects of antibiotics were likely small during the study period.

## Conclusion

Decreased diversity of the airway microbiome, increased abundance of *Stenotrophomonas*, and increased level of sn-glycerol 3-phosphoethanolamine might have potential as biomarkers for BPD. The occurrence and severity of BPD are closely related to *Stenotrophomonas*, which might influence the composition of the lower airway microbiome through its metabolite sn-glycerol 3-phosphoethanolamine, and might be the triggering factor of the disease. The causal relationship needs further study.

## Data Availability Statement

Sequence data presented in the study are publicly available. This data can be found here: (http://www.ncbi.nlm.nih.gov/sra/, PRJNA800242).

## Ethics Statement

The study involving human participants was reviewed and approved by the Institutional Review Board of Chongqing Medical University. Written informed consent to participate in this study was provided by the participants’ legal guardian/next of kin.

## Author Contributions

QX and JY conceived and designed the study, collected the data, drafted the initial manuscript, and reviewed and revised the manuscript. DL designed the data collection instruments, collected data, carried out the initial analyses, and reviewed and revised the manuscript. QT and YH conceived and designed the study, coordinated and supervised data collection, and critically reviewed the manuscript for important intellectual content. All authors approved the final manuscript as submitted and agreed to be accountable for all aspects of the work.

## Conflict of Interest

The authors declare that the research was conducted in the absence of any commercial or financial relationships that could be construed as a potential conflict of interest.

## Publisher’s Note

All claims expressed in this article are solely those of the authors and do not necessarily represent those of their affiliated organizations, or those of the publisher, the editors and the reviewers. Any product that may be evaluated in this article, or claim that may be made by its manufacturer, is not guaranteed or endorsed by the publisher.

## Footnotes

1
http://www.genome.jp/kegg/pathway.html
