# Supplementary material for: The Airway Microbiome and Metabolome in Preterm Infants: Potential Biomarkers of Bronchopulmonary Dysplasia
Source: Front Pediatr. 2022 May 10;10:862157. doi: 10.3389/fped.2022.862157 (PMC9127389; doi:10.3389/fped.2022.862157)

**
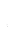

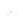

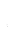

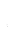
Supplement Table 1 Parameters of the PCA models of metabolomics data.**

| **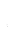groups** | **number of principal components** | **R2X(cum)** |
| --- | --- | --- |
| 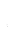birth [Day 1] ES+ | 4 | 0.584 |
| 7 days after birth ES+ | 6 | 0.812 |
| birth [Day 1] ES- | 5 | 0.638 |
| 7 days after birth ES- | 3 | 0.588 |


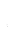

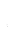

Supplement: Supplementary file 1 [file Table_1.DOCX]
